# Forcing dividing cancer cells to die; low‐dose drug combinations to prevent spindle pole clustering

**DOI:** 10.1007/s10495-021-01671-3

**Published:** 2021-04-19

**Authors:** Eloise Ducrey, Cédric Castrogiovanni, Patrick Meraldi, Patrycja Nowak-Sliwinska

**Affiliations:** 1 School of Pharmaceutical Sciences, Faculty of Sciences, University of Geneva, Rue Michel-Servet 1, CMU, 1211 Geneva 4, Switzerland; 2 Institute of Pharmaceutical Sciences of Western Switzerland, University of Geneva, Rue Michel-Servet 1, CMU, 1211 Geneva 4, Switzerland; 3 Translational Research Center in Oncohaematology, Rue Michel-Servet 1, CMU, 1211 Geneva 4, Switzerland; 4 Department of Cell Physiology and Metabolism, University of Geneva Medical School, Rue Michel-Servet 1, CMU, 1211 Geneva 4, Switzerland

**Keywords:** Mitosis, Drug combination, Cancer, Centrosome clustering

## Abstract

Mitosis, under the control of the microtubule-based mitotic spindle, is an attractive target for anti-cancer treatments, as cancer cells undergo frequent and uncontrolled cell divisions. Microtubule targeting agents that disrupt mitosis or single molecule inhibitors of mitotic kinases or microtubule motors kill cancer cells with a high efficacy. These treatments have, nevertheless, severe disadvantages: they also target frequently dividing healthy tissues, such as the haematopoietic system, and they often lose their efficacy due to primary or acquired resistance mechanisms. An alternative target that has emerged in dividing cancer cells is their ability to “cluster” the poles of the mitotic spindle into a bipolar configuration. This mechanism is necessary for the specific survival of cancer cells that tend to form multipolar spindles due to the frequent presence of abnormal centrosome numbers or other spindle defects. Here we discuss the recent development of combinatorial treatments targeting spindle pole clustering that specifically target cancer cells bearing aberrant centrosome numbers and that have the potential to avoid resistance mechanism due their combinatorial nature.

Mitosis is a well-regulated physiological process of cell division that equally distributes genetic material into two identical daughter cells. Faithful chromosome segregation relies on the bipolar mitotic spindle, composed of dynamic microtubules. When cells enter mitosis, the duplicated microtubules organizing centre, called centrosomes, migrate towards opposite sides of the nucleus and catalyse the assembly of the bipolar spindle as the nuclear envelope breaks down. Microtubules bind and align the sister chromatids on the metaphase plate, before pulling them apart towards poles in anaphase. To ensure proper chromosome segregation and avoid chromosomal instability, as well as aneuploidy [1, 2], the spindle assembly checkpoint (SAC) delays anaphase onset until all chromosomes are attached to spindle poles [3, 4].

Mitosis is an ideal target for anti-cancer treatments since uncontrolled cell proliferation is a hallmark of cancer [5] and since every cancer cell must divide to proliferate. During mitosis cells are in fragile conditions: chromosomes are hyper-condensated and prone to breakage or loss, and most transcription has been turned off. It is therefore not surprising that disruption of the mitotic process can lead to cell death [6]. Faithful mitotic progression relies on correct microtubule dynamics, their intrinsic ability to shrink and grow, allowing them to explore the three-dimensional space and to generate mechanical forces. Therefore, microtubule-targeting agents have emerged early on as classical tools to target dividing cancer cells. Impairment of correct microtubule dynamics can disrupt kinetochore-microtubule attachment, preventing SAC satisfaction, and resulting in a prolonged mitotic arrest. Such a mitotic arrest rapidly leads to a caspase-dependent cell death [7]. Alternatively, cells with massive chromosome segregation defects can undergo cell death in the ensuing interphase due the loss of essential genes [8–10].

Microtubule-destabilizing drugs, such as vinblastine, prevent SAC satisfaction, causing mitotic cell death. Microtubule-stabilizing drugs, such as paclitaxel (Taxol^®^) or epothilone B, stimulate microtubule polymerization and promote the formation of multipolar spindles [9]. Multipolar cell divisions result in unbalanced chromosome segregation and aneuploidy in daughter cells, causing cell death in the next interphase [8, 9, 11, 12]. Taxanes are still amongst the most successful chemotherapeutic agents used in the clinic, in particular to treat breast, prostate or ovarian cancers [8].

Microtubule-targeting agents induce, however, severe side effects. By targeting dividing cells, those drugs impact any healthy tissues displaying a high rate of cell division, in particular the hematopoietic lineage and the digestive system. In addition, those agents also affect cells that depend on microtubules dynamics in interphase, such as neurons or endothelial cells. This, in turn, results in severe pathologies affecting amongst others, blood cell levels, the immune system, and painful neuropathies in the extremities [7]. Moreover, a substantial number of patients do not respond to these treatments, as they carry a primary resistance or they will rapidly develop acquired resistance to those microtubule-targeting agents [13].

The second type of anti-mitotic drugs targets specific mitotic kinases (e.g. Aurora or Polo-like kinases) or microtubule-motor proteins (e.g. the kinesin-5 KIF11/Eg5), in order to prevent mitotic progression while avoiding the detrimental effects of microtubule-targeting agents. However, the clinical use of these mitotic inhibitors did neither provide an improvement over the standard of care, due to their high toxicity towards the hematopoietic system, nor did it prevent early development of acquired resistances. Additionally, the activity of above-mentioned drugs might very well depend on a cancer type that would be identified as sensitive to those drugs [8, 14]. Therefore, there is still an unmet need to identify anti-cancer agents that specifically and safely target dividing cancer cells.

Another attractive anti-mitotic strategy is to target centrosome abnormalities. Supernumerary centrosomes are frequent in human cancer cells [15, 16] and can lead to the formation of multipolar spindles. Persistent multipolarity will result in lethal multipolar divisions [10, 17, 18], see Fig. 1. To avoid such a death, cancer cells use a survival mechanism called spindle pole clustering, in which they cluster their extra spindle poles onto two spindle poles to achieve a pseudo-bipolar cell division [19, 20].

**Fig. 1:**
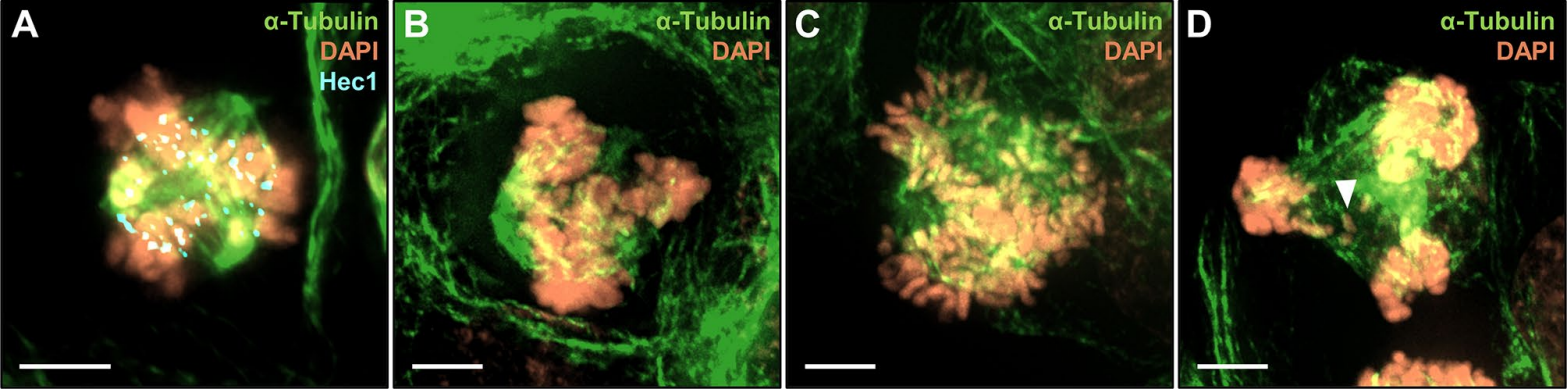
Representative immunofluorescence images of multipolar cells. **a** HCT-116 human colorectal carcinoma cell treated with 3 nM of Taxol for 16 h (Hec1 is used as a marker for kinetochores). **b**–**d** HT29 human colorectal adenocarcinoma cells treated with 3 nM of Taxol for 16 h. **d** Representative image of a HT29 multipolar cell in late anaphase. Arrow shows a lagging chromosome. Scale bar = 5 μm

Spindle pole clustering depends on a number of forces acting on the mitotic spindle. These include the forces generated by the minus-end directed microtubule-motor proteins dynein and kinesin-14 HSET, and forces emanating from the stiffness of the cell cortex [21]. Proof-of-principle experiments indicated that HSET depletion impaired spindle pole clustering and led to lethal multipolar divisions in cancer cells with extra-centrosomes, while leaving non-cancer cells with normal centrosome numbers unaffected [21]. This specificity confirms that spindle pole clustering provides a window of opportunity for anti-cancer drugs that inhibit this process.

In theory, this window of opportunity goes beyond cancer cells with an elevated number of centrosomes, since extra spindle poles can also arise *via* other pathways, i.e. (i) after a centrioles dis-engagement, resulting in additional spindle poles with only one centriole, (ii) following an exceedingly long centriole breakage, leading to higher number of spindle poles [16] or (iii) when cells form extra acentrosomal poles due to a force imbalance within the mitotic spindle [19, 20].

Examples of anti-cancer drugs acting *via* multipolar spindles are Taxol^®^ or epothilone B, both known to induce multipolarity [22]. Another drug to point out is a platelet-derived growth factor receptor β (PDGFR-β) inhibitor, crenolanib, which prevents pole clustering [23]. Interestingly, we and others have shown that crenolanib induces mitotic spindle multipolarity by activating the actin-severing protein cofilin, leading to destabilization of the cortical actin network, instead of acting via PDGFR-β inhibition [23, 24].

Unfortunately, their use as individual drugs invariably leads to acquired resistance and severe side effects in treated patients. Currently, multiple single-molecule inhibitors targeting spindle pole clustering are in pre-clinical development [25], but they are likely to lead to the development of resistances in cancer patients, as previously observed for other single anti-mitotic inhibitors.

One promising approach that could resolve above-listed issues is to replace a single anti-cancer agent administered at high doses by a synergistic combination of several drugs applied at low-doses that target various signalling pathways specifically in cancer cells. Such combinatorial treatments present milder side-effects, since the individual drugs are used at concentrations well below the maximally tolerated doses used in the clinical setting [26–30]. Using our validated phenotypic Therapeutically Guided Multidrug Optimization method we optimized a low-dose drug combination (ODC). This drug cocktail consists of two tyrosine kinase inhibitors (erlotinib and dasatinib) and two histone deacetylase inhibitors (tacedinaline and tubacin), for which we observed dose-dependent synergistic drug-drug interactions. This methodology samples a minimal number of experimental data points to create the response surface to drug combinations in terms of second-order linear regression models that are used to select synergistic drug combinations [31, 32]. The activity of our optimized drug combination on cancer cell viability correlates with its ability to induce multipolar spindle formation and inhibition of multipolar spindle clustering [33], whereas none of the four monotherapies affected pole clustering on their own at the tested dose range, see Fig. 2. Our optimized drug combination targets with high efficacy renal cell carcinoma, colorectal carcinoma or melanoma, while showing negligible toxicity towards non-malignant organ-specific epithelial cells.

**Fig. 2:**
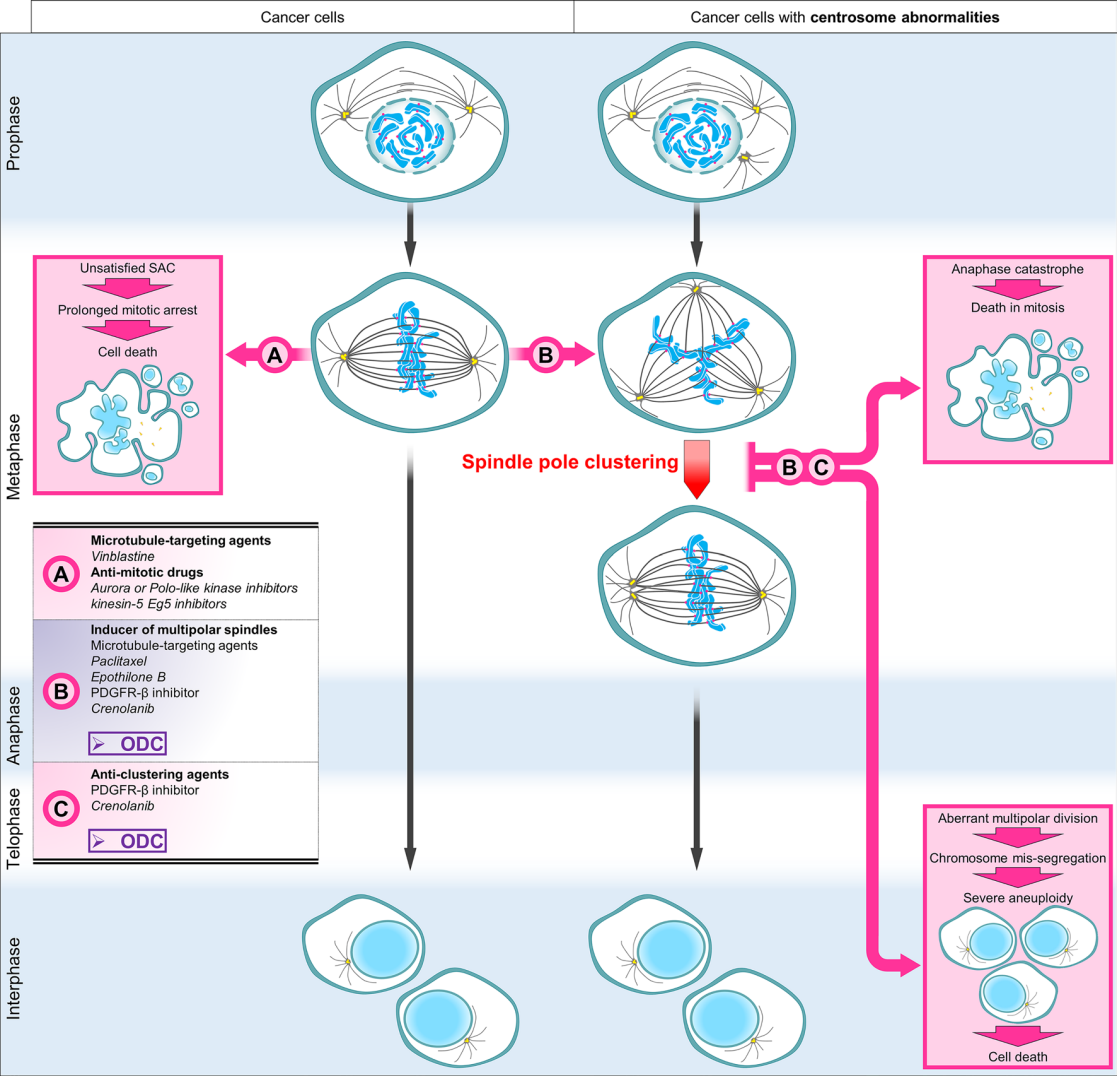
Therapeutic strategies to target mitosis in cancer. **a**–**c** represent the three main axes of therapeutic strategies used to kill dividing cancer cells. On the left, division of cancer cells with normal number of centrosomes. On the right, division of cancer cells with centrosome abnormalities. Those cells are prone to form multipolar spindles and to cluster their extra spindle poles to achieve pseudo-bipolar cell division. Compared to current anti-mitotic drugs (**a** and **b**), the optimized drug combination (ODC) can target two different axes of mitosis in cancer cells, while reducing the risk of acquired resistance and serious side effects. ODC acts both on cancer cells with and without centrosome abnormalities by inducing the formation of multipolar spindles (**b**). In addition, ODC inhibits spindle pole clustering of cancer cells (**b** and **c**). This leads to cell death in mitosis or in the following interphase due to severe chromosome mis-segregations

Summarizing, it is being widely recognized that the inhibition of spindle pole clustering is a promising therapeutic strategy that specifically leads to cancer cell death, while leaving non-malignant cells with normal number of centrosomes unaffected. Multipolar spindles are present in several cancers and force cancer cells to cluster their poles to achieve a pseudo-bipolar cell division and survive. Since this vulnerability is absent in normal dividing cells in the body, it is an interesting Achilles heel to target.
